# The Association of Distinct Social Determinants of Health with Added Sweetener Knowledge and Consumption in a U.S. Sample of People Living with HIV

**DOI:** 10.1007/s10461-021-03508-1

**Published:** 2021-11-03

**Authors:** Emma M. Kileel, Kirsten A. Dickins, Hui Zheng, Kathleen V. Fitch, Sara E. Looby

**Affiliations:** 1 Metabolism Unit, Massachusetts General Hospital, Boston, MA USA; 2 Yvonne L. Munn Center for Nursing Research, Massachusetts General Hospital, Boston, MA USA; 3 Biostatistics Center, Massachusetts General Hospital, Boston, MA USA; 4 Metabolism Unit, Massachusetts General Hospital and Harvard Medical School, Boston, MA USA; 5 Metabolism Unit and the Yvonne L. Munn Center for Nursing Research, Massachusetts General Hospital and Harvard Medical School, 55 Fruit Street LON 207, Boston, MA 02114 USA

**Keywords:** HIV, Sweetening agents, Social determinants of health

## Abstract

Prior studies in people living with HIV (PLWH) suggest added sweetener intake exceeds recommendations and associates with cardiometabolic abnormalities. Little is known of factors that associate with increased sweetener consumption in PLWH. This cross-sectional study explored knowledge and consumption of added sweeteners and associations of social determinants of health (SDoH) among 900 PLWH residing in the United States. Demographics, SDoH, and added sweetener knowledge and consumption were assessed via an online survey, multivariable analyses were completed. Results demonstrate that sex, race, and low educational level associated with lower sweetener knowledge. Race, age, body mass index, income, limited access to fresh fruits/vegetables, and low sweetener knowledge associated with higher sweetener consumption. Findings highlight the need for consideration of specific demographics and inequitable social circumstances when developing nutrition lifestyle strategies, inclusive of added sweetener education, that are feasible and sustainable across diverse community settings of PLWH.

**Supplementary Information:**

The online version contains supplementary material available at 10.1007/s10461-021-03508-1.

## Resumen

Estudios previos en personas que viven con el VIH (PLWH) sugieren que la ingesta adicional de edulcorantes excede las recomendaciones y se asocia con anomalías cardiometabólicas. Poco se sabe de los factores que se asocian con un mayor consumo de edulcorantes en PLWH. Este estudio transversal exploró el conocimiento y el consumo de edulcorantes agregados y las asociaciones de los determinantes sociales de la salud (SDoH) entre 900 PLWH que residen en los Estado Unidos. Lo datos demográficos, SDoH, y en conocimiento y el consumo de edulcorantes añadidos se evaluaron mediante una encuesta en línea y se completaron análisis multivariables. Los resultados demuestran que el sexo, la raza y el bajo nivel educativo se asocian con un menor conocimiento sobre edulcorantes. Raza, edad, índice de masa corporal, ingresos, acceso limitado a frutas/verduras frescas y conocimiento bajo sobre edulcorantes asociado con un mayor consumo edulcorantes. Los hallazgos resaltan la necesidad de considerar la demografía específica y las circunstancias sociales desiguales al desarrollar estrategias de estilo de vida nutricional, incluida la educación adicional sobre edulcorantes, que sean factibles y sostenibles en diversos entornos comunitarios de PLWH.

## Introduction

Weight gain and obesity have emerged as contemporary health concerns in people living with HIV (PLWH) engaged in treatment [1–3] and may in part be related to poor diet and increased consumption of added sweeteners. In the United States, increased intake of added sweeteners, both naturally occurring and artificial, may influence the development of adverse cardiometabolic conditions including obesity, diabetes, and cardiovascular disease (CVD) [4–8].

Studies pertinent to HIV and sweeteners are limited, though findings suggest that sweetener consumption is increased among PLWH compared to people without HIV, and negative clinical correlates are associated with sweetener consumption in PLWH. Hall et al. demonstrated that PLWH, compared to adults without HIV, had higher intake of total sugar and added sugar, as well as the artificial sweetener, aspartame, which was associated with coronary plaque [9]. Tiozzo et al. showed that PLWH with metabolic syndrome consumed 19% of daily calories from added sugar and PLWH without metabolic syndrome consumed 15% of daily calories from added sugar. In this study, both groups (PLWH with and without metabolic syndrome) exceeded added sugar intake recommendations, suggesting that increased intake of added sugar may contribute to hyperglycemia and higher rates of metabolic syndrome in PLWH [10]. Findings from a large cohort study of women with HIV suggested a higher percentage of total daily caloric intake from “sweets” (including cookies, cakes, candy, sweetened cereals) was associated with increased insulin resistance [11]. However, these studies did not explore factors that might influence added sweetener consumption in PLWH, nor did they examine if lack of knowledge about added sweeteners associates with sweetener consumption.

Many PLWH experience disproportionate social and economic burden [12], and demonstrate sub-optimal adherence to dietary guidelines [13]. Understanding factors that influence added sweetener knowledge and consumption in PLWH may help inform educational curriculums integrated into nutritional interventions for this population. The primary objective of this cross-sectional survey study was to explore added sweetener knowledge and consumption in a U.S. sample of PLWH. As a sub-objective, we investigated factors which may associate with added sweetener knowledge and consumption including social determinants of health (SDoH).

## Methods

### Design and Participants

An anonymous online survey was conducted among a U.S. sample of PLWH between February 2019 and January 2020. A variety of recruitment strategies were employed to ensure outreach to PLWH of diverse sex, race, and ethnicity. Participants were recruited in the United States via an advertisement that included a link to the study survey. Upon accessing the survey, participants were prompted to attest to eligibility criteria including age at least 18 years, residing in the United States, and being HIV positive (see Supplemental Material 1 for the participant flow chart). Study advertisements in English and Spanish were posted on online platforms for organizations and publications known to the HIV community including: *The Well Project*, *Adelante*, *Positively Aware*, *The Body*, and *HIV Plus*; and social media: *Twitte*r, *Facebook*, and *Craigslist*. Other outreach strategies included a collaboration with *The Henne Group*, an organization that distributes email communications with study information to a panel of PLWH, and a mass-mailing of study postcards and flyers that included the survey link and a scannable quick response (QR) code sent to over 40 HIV community organizations, health centers, and hospitals throughout the United States. People living with HIV in attendance at health fairs or meetings at HIV community organizations proximal to the study investigators in Massachusetts were also invited to complete the survey via tablet.

### Measures

#### Survey Development

The survey was developed by the study investigators using adapted versions of previously published valid and reliable questionnaires to assess: demographic and clinical characteristics, SDoH [14], sweetener knowledge [15], and sweetener consumption [16–18]. For this study, added sweeteners included both naturally occurring sugars and artificial sweeteners (aspartame, saccharin, sucralose, among others). Prior to survey development, a focus group consisting of a diverse community sample of PLWH was conducted to solicit feedback on the survey content for clarity and length, online usability, and appeal of study advertisements [19]. Based on findings from the focus group, the final survey was refined to 33 multiple choice and open-ended questions (Supplemental Material 2).

The survey was hosted on the web-based platform *Partners Rally*, developed by Partners HealthCare Research and the Massachusetts General Hospital Laboratory of Computer Science (https://rally.partners.org/about). The survey was available in English and Spanish. All data were collected by participant self-report.

#### Sweetener Knowledge and Consumption

Consistent with methods from a prior sweetener knowledge study in people without HIV [15], a cumulative total sweetener knowledge score was established. Sweetener knowledge was assessed via 11 questions, correct answers were assigned a value of 1 and incorrect answers were assigned value of 0. The total possible sweetener knowledge score ranged from 0 to 11, with a higher score indicating greater knowledge. For analysis purposes, sweetener knowledge score was treated as a continuous variable.

Sweetener consumption was assessed via 13 questions. Continuous total sweetener consumption scores were computed for the following: total food consumption, total drink consumption, and total consumption (which included total food plus total drink consumption). Participants were provided a list of food and drink items and prompted to rate consumption as *usually/often* (weighted as 2), *sometimes* (weighted as 1), or *rarely*/*never* (weighted as 0). Scores were summed and the total possible score range for each variable was: total food consumption 0–12, total drink consumption 0–14, and total consumption 0–26; higher scores indicated greater consumption. For analysis purposes sweetener consumption score was treated as a continuous variable.

#### Demographic, Social, and Clinical Characteristics

Demographic and HIV clinical data including age (years), sex at birth, gender identity, race, ethnicity, duration of HIV (years), co-morbid conditions, current antiretroviral therapy (ART) use, and HIV viral load (detectable/undetectable) were collected (Supplemental Material 2). Additionally, SDoH were assessed, specifically, health literacy, food security, housing stability, and financial resources [14]. Participants were asked to report current weight and height, and body mass index (BMI) was calculated using standard calculations and assessed according to established obesity parameters [20]. The survey item related to ART adherence was adapted from the Center for Adherence Support Evaluation Adherence Index [21].

#### Study Outcomes and Covariates

The study outcomes were respondent knowledge and consumption of added sweeteners. The association of SDoH, age, sex, race, and BMI with added sweetener knowledge and consumption was evaluated.

### Statistical Analysis

The Shapiro–Wilk test was used to assess normality; non-normally distributed continuous variables are presented as median (lower quartile [Q1], upper quartile [Q3]) and categorical variables as frequencies and percentages.

Univariate analyses were performed to evaluate the relationship between explanatory variables including: sex, race, educational attainment, age, health literacy (“Can you understand health information provided from a doctor, nurse or clinic?”, *yes/ no*), and sweetener knowledge score using a linear regression model. Univariate analyses were also conducted to assess the relationship between the following variables: sex, race, age, BMI, income, food security (“Do you have access to fresh fruit and vegetables?”, *yes/no*), and sweetener consumption score using a linear regression model.

Variables with significant associations with each outcome in the univariate models were selected for placement in multivariable models. The variable, “Can you understand the health information provided from your doctor, nurse or clinic*?”* was excluded from the multivariable model for total sweetener knowledge due to evidence of multicollinearity between this variable and educational attainment. Additionally, 99% of respondents answered “*Yes”* to this question. For the purpose of the multivariable analyses race represents Black or African American, Asian, American Indian or Alaskan Native, Native Hawaiian or other Pacific Islander, more than one race, or other, education represents < high school diploma or GED versus ≥ high school diploma or GED, annual income represents < $25,000 versus ≥ $25,000 based on 2020 Poverty Guidelines [22] and the variable “access to fresh fruits and vegetables” was grouped to *all of the time/some of the time* versus *never*. Multivariable linear regression modeling was performed with sweetener knowledge score and sweetener consumption score as separate dependent variables and 95% confidence intervals were computed to determine significant independent associations within each model. Data were downloaded from *Partners Rally* to Excel® and exported to JMP® Pro 13 (Cary, NC) statistical software for analysis, inference was based on a nominal significance level (p ≤ 0.05).

#### Power Calculation

For the primary study outcome, it was estimated that with a minimum of 800 participants, we would be able to construct a 95% confidence interval for the true proportion of correct responses regarding added sweetener knowledge with a width of 0.03 (i.e., 0.32 ± 0.03) = (0.29, 0.35). Nine-hundred respondents completed the survey.

## Results

### Demographic and Clinical Characteristics

**Fig. 1:**
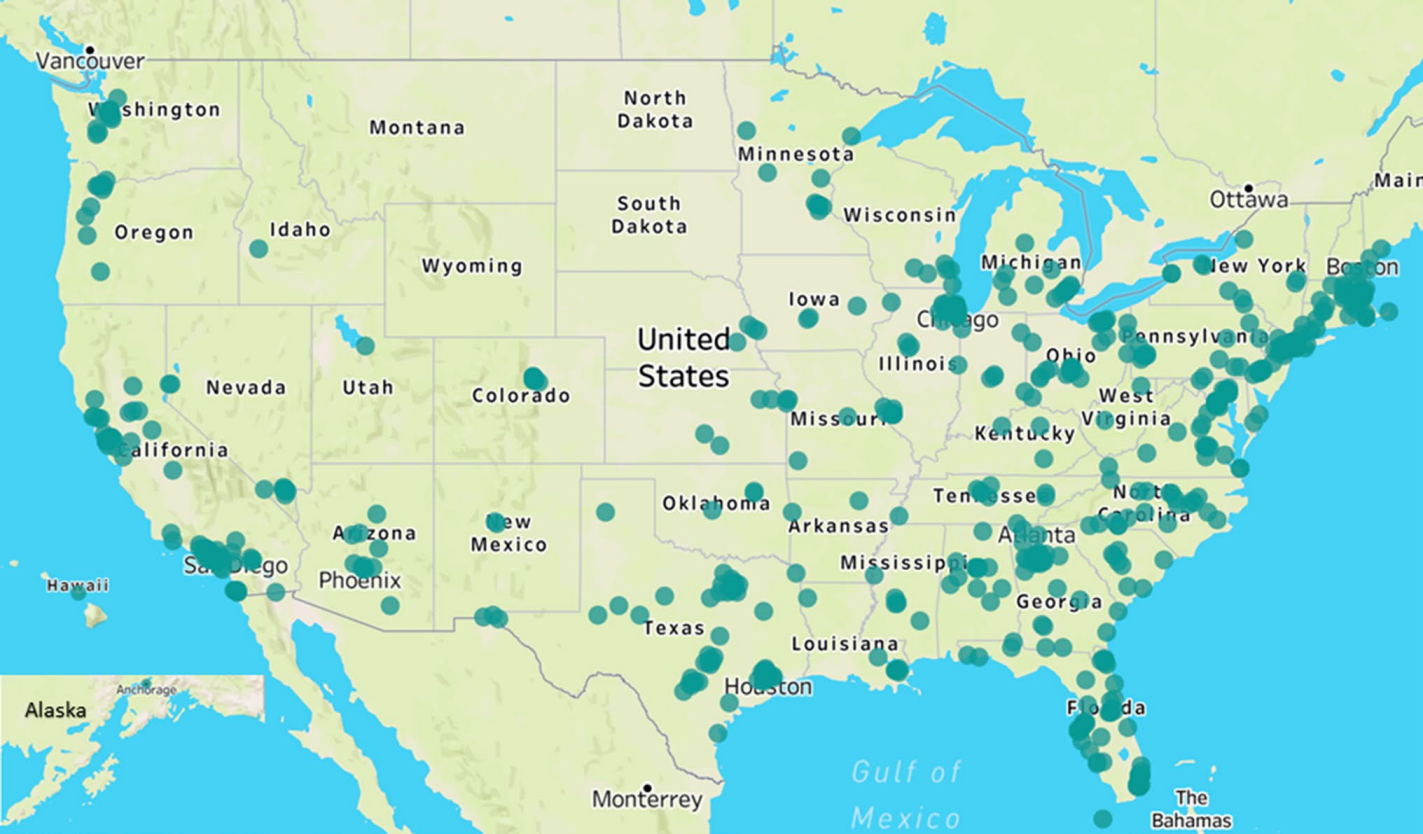
Location of residence of respondent by zip code. Alaska and Hawaii moved for illustrative purposes. Missing zip codes = 43

Respondents resided in 46 of 52 U.S. states and territories; Fig. 1 illustrates location of residence by zip code. Demographic and clinical characteristics of the survey respondents are summarized in Table 1. The median age of the respondents was 54 years (lower quartile [Q1], upper quartile [Q3]: 44, 60 years), 80% reported male natal sex, 97% identified as cisgender, 22% were Black or African American, 13% identified as Hispanic/Latino. Regarding clinical characteristics, 71% were overweight or obese, the median number of years living with HIV was 18 (9, 27), 93% of respondents reported having an undetectable HIV viral load and 98% reported current treatment with ART. The median number of comorbidities (hypertension, hyperlipidemia, cancer, CVD, pre-diabetes, diabetes, asthma) reported among the respondents was 1 (0, 2).

**Table 1:** Demographic and clinical characteristics

	*n* (%) or median (Q1, Q3)
	Total *n* = 900
Age (years)	54 (44, 60)
Natal sex	
Male	724 (80%)
Gender identity*	
Cisgender	867 (97%)
Non-cisgender	29 (3%)
Race	
Black or African American	194 (22%)
White	609 (68%)
Asian	8 (< 1%)
American Indian or Native Alaskan	8 (< 1%)
Native Hawaiian or other Pacific Islander	2 (< 1%)
Other	41 (5%)
More than one race	38 (4%)
Hispanic ethnicity	114 (13%)
BMI (kg/m^2^)	27.4 (24.3, 31.7)
BMI category^¥^	
< 25 kg/m^2^	261 (29%)
≥ 25 – 30 kg/m^2^ (overweight)	337 (38%)
≥ 30 kg/m^2^ (obese)	295 (33%)
Number of comorbidities	1 (0, 2)
Duration of HIV (years)	18 (9, 27)
Undetectable HIV viral load	839 (93%)
Current ART use	878 (98%)

*ART* antiretroviral therapy, *BMI* body mass index

*Non-cisgender includes participants identifying as transgender, gender queer, gender variant, and ‘other’. Missing gender identity data, *n* = 4

^¥^7 BMI data points not included: Missing data, *n* = 5 Outlier data, *n* = 2 removed due to erroneous height and weight data that yielded inaccurate BMI calculation [2.35 kg/m^2^ and 10.18 kg/m^2^]

^±^Missing ART data, *n* = 22

### Social Determinant of Health Characteristics

Table 2 summarizes SDoH characteristics. Sixty-five percent of respondents reported educational attainment beyond high school/GED, and 64% of respondents reported an annual income between $1 and $49,999. Over 90% of respondents reported current health insurance enrollment, stable housing, and the ability to understand information provided by a healthcare provider.

**Table 2:** Social determinants of health characteristics

	*n* (%)
	Total *n* = 900
Highest level of education	
Less than a high school/GED	17 (2%)
High school/GED	294 (33%)
Post high school/GED	589 (65%)
Income level	
$1–$49,999	572 (64%)
$50,000–$99,999	212 (24%)
$100,000 and greater	116 (13%)
Reported health insurance	
Yes	850 (94%)
Reported stable housing	
Yes	854 (95%)
Can you understand the health information provided from your doctor, nurse or clinic?	
Yes	889 (99%)
In the last year did you ever eat less than you felt you should because there wasn’t enough money for food?	
Yes	236 (26%)
Do you have access to fresh fruit and vegetables?	
All of the time?	608 (68%)
Some of the time?	278 (31%)
Never?	14 (2%)
Do you have a place to store and cook food?	
All of the time?	843 (94%)
Some of the time?	51 (6%)
Never?	6 (< 1%)

*GED* graduate equivalency degree

Responses pertaining to food security are presented in Table 2. Approximately one quarter of respondents reported eating less in the last year because there was not enough money for food. Similarly, 31% of respondents reported having access to fresh fruits and vegetables only *some of the time* versus *all of the time* or *never*.

### Sweetener Knowledge and Consumption

Sweetener knowledge and consumption scores are shown in Table 3. Median sweetener knowledge score was 8 (7, 9) of a total possible score range of 0–11. The median total sweetener consumption score was 9 (6, 12). Evaluating sweetener consumption delineated by food and drink intake, median total food consumption score was 5 (4, 7), and median total drink consumption score (excluding alcohol) was 3 (2, 5).

**Table 3:** Sweetener knowledge and consumption scores

	Median (Q1, Q3)
	Total *n* = 900
Sweetener Knowledge Score*	
Total knowledge score	8 (7, 9)
Sweetener Consumption Scores^¥^	
Total consumption score	9 (6, 12)
Total food consumption score	5 (4, 7)
Total drink consumption score	3 (2, 5)

*Possible knowledge score range 0–11, with a higher score indicating greater knowledge

^¥^Possible consumption score range 0–26. Possible food consumption score: 0–12. Possible drink consumption score (without alcohol): 0–14. Higher scores indicate greater consumption

### Associations with Demographics, Social Determinants of Health, and Sweetener Knowledge

In univariate analyses (Table 4a), female sex (β estimate − 0.41; 95% CI − 0.55, − 0.26; F ratio 30.59; p < 0.0001), race (β estimate − 0.75; 95% CI − 0.86, − 0.63; F ratio 163.46; p < 0.0001), having less than a high school diploma/GED (β estimate − 0.92; 95% CI − 1.34, − 0.49; F ratio 18.02; p < 0.0001), and not understanding information provided by a health provider or clinic (β estimate − 0.97; 95% CI − 1.49, − 0.44; F ratio 13.00; p = 0.0003) were negatively associated with sweetener knowledge score, while age (β estimate 0.02; 95% CI 0.01, 0.03; F ratio 15.71; p < 0.0001) was positively associated with sweetener knowledge score. In the multivariable model (Table 4a), female sex (β estimate − 0.16; 95% CI − 0.30, − 0.02; F ratio 5.19; p = 0.02), race (β estimate − 0.68; 95% CI − 0.80, − 0.56; F ratio 120.78; p < 0.0001), and having less than a high school diploma/GED (β estimate − 0.61; 95% CI − 1.00, − 0.21; F ratio 9.00; p = 0.003) each remained significantly associated with lower total sweetener knowledge score (*R*^2^ = 0.17; F ratio 46.06; p < 0.0001).

**Table 4:** Univariate and multivariable regression models of added sweetener knowledge and added sweetener consumption scores

a. Associations with sweetener knowledge score												
Characteristics	Univariate						Multivariable					
							(R^2^ = 0.17; F ratio = 46.06; p < 0.0001)^*^					
	β-Estimate		95% CI	Test statistic^Ŧ^		p-Value	β-Estimate	95% CI		Test statistic^Ŧ^		p-Value
Female sex	− 0.41	[− 0.55, − 0.26]		30.59	< 0.0001		− 0.16	[− 0.30, − 0.02]		5.19		0.02
Race^¥^	− 0.75	[− 0.86, − 0.63]		163.46	< 0.0001		− 0.68	[− 0.80, − 0.56]		120.78		< 0.0001
Less than a high school degree or GED^¥^	− 0.92	[− 1.34, − 0.49]		18.02	< 0.0001		− 0.61	[− 1.00, − 0.21]		9.00		0.003
Age (years)	0.02	[0.01, 0.03]		15.71	< 0.0001		0.01	[− 0.00, 0.01]		1.37		0.24
Can you understand the health information provided from your doctor, nurse or clinic? (no)	− 0.97	[− 1.49, − 0.44]		13.00	0.0003							

b. Associations with sweetener consumption score									
Characteristics	Univariate					Multivariable			
						(R^2^ = 0.21; F ratio = 33.67; p < 0.0001)^*^			
	β-Estimate		95% CI	Test statistic^Ŧ^	p-Value	β-Estimate	95% CI	Test statistic^Ŧ^	p-Value
Female sex	0.75	[0.37, 1.13]		15.27	0.0001	− 0.04	[− 0.41, 0.33]	0.04	0.83
Race^¥^	1.37	[1.05, 1.68]		74.45	< 0.0001	0.53	[0.20, 0.87]	9.77	0.002
Age (years)	− 0.12	[− 0.14, − 0.09]		88.88	< 0.0001	− 0.10	[− 0.12, − 0.07]	62.39	< 0.0001
BMI (kg/m^2^)	0.07	[0.03, 0.11]		10.26	0.001	0.05	[0.01, 0.09]	5.04	0.03
Annual income less than $25,000^¥^	1.00	[0.69, 1.31]		41.22	< 0.0001	0.69	[0.40, 0.99]	21.24	< 0.0001
Access to fresh fruits and vegetables? (never) ^¥^	3.49	[2.29, 4.69]		32.56	< 0.0001	2.73	[1.58, 3.87]	21.88	< 0.0001
Sweetener knowledge score	− 0.73	[− 0.89, − 0.57]		77.29	< 0.0001	− 0.43	[− 0.60, − 0.26]	24.51	< 0.0001

*CI* confidence interval, *GED* graduate equivalency degree

^Ŧ^Associated test statistic for p-Value = F ratio

*R^2^ represents the coefficient of determination and the proportion of variance explained by the model, p-Value represents significance by the whole model ANOVA test

^¥^For the purpose of the multivariable analyses race represents Black or African American, Asian, American Indian or Alaskan, Native Hawaiian or other Pacific Islander, more than one race, or other, education (< high school diploma or GED versus ≥ high school diploma or GED), annual income (< $25,000 versus ≥ $25,000 based on 2020 Poverty Guidelines https://aspe.hhs.gov/poverty-guidelines), and access to fresh fruits and vegetables (all of the time/some of the time versus never)

### Associations with Demographics, Social Determinants of Health, and Sweetener Consumption

In the univariate analyses (Table 4b), female sex (β estimate 0.75; 95% CI 0.37, 1.13; F ratio 15.27; p = 0.0001), race (β estimate 1.37; 95% CI 1.05, 1.68; F ratio 74.45; p < 0.0001), BMI (β estimate 0.07; 95% CI 0.03, 0.11; F ratio 10.26; p = 0.001), annual income < $25,000 (β estimate 1.00; 95% CI 0.69, 1.31; F ratio 41.22; p < 0.0001), and never having access to fresh fruits and vegetables (β estimate 3.49; 95% CI 2.29, 4.69; F ratio 32.56; p < 0.0001) were all positively associated with total sweetener consumption score. Sweetener knowledge (β estimate − 0.73; 95% CI − 0.89, − 0.57; F ratio 77.29; p < 0.0001) (Supplemental Material 3) and age (β estimate − 0.12; 95% CI − 0.14, − 0.09; F ratio 88.88; p < 0.0001) were negatively associated with sweetener consumption score. In the multivariable analysis (Table 4b), race (β estimate 0.53; 95% CI 0.20, 0.87; F ratio 9.77; p = 0.002), age (β estimate − 0.10; 95% CI − 0.12, − 0.07; F ratio 62.39; p < 0.0001), BMI (β estimate 0.05; 95% CI 0.01, 0.09; F ratio 5.04; p = 0.03), annual income < $25,000 (β estimate 0.69; 95% CI 0.40, 0.99; F ratio 21.24; p < 0.0001), never having access to fresh fruits and vegetables (β estimate 2.73; 95% CI 1.58, 3.87; F ratio 21.88; p < 0.0001), and low sweetener knowledge score (β estimate − 0.43; 95% CI − 0.60, − 0.26; F ratio 24.51; p < 0.0001) all remained significant predictors of total sweetener consumption (*R*^2^ = 0.21; F ratio 33.67; p < 0.0001).

Sensitivity analyses were performed for each outcome (sweetener knowledge and sweetener consumption), including the variable, *told by a healthcare provider you have diabetes* (*yes*: *n* = 134/900; 15%) in each multivariable model. Having diabetes was not a significant predictor of sweetener knowledge (β estimate 0.01; 95% CI − 0.14, 0.17; F ratio 0.03; p = 0.87) or sweetener consumption (β estimate 0.33; 95% CI − 0.06, 0.73; F ratio 2.84; p = 0.09).

## Discussion

In a geographically diverse, U.S.-based sample of 900 PLWH, we demonstrate that key demographics and SDoH are important factors associated with added sweetener knowledge and consumption. Factors including sex, race, age, BMI, education, income, and access to fresh fruits and vegetables associated with lower sweetener knowledge and higher sweetener consumption in PLWH. Further, lower sweetener knowledge was associated with higher sweetener consumption. These findings highlight the need for consideration of these specific factors when developing patient-centered nutrition lifestyle strategies that are feasible and sustainable across diverse community settings of PLWH.

In our study, lower educational attainment correlated with lower sweetener knowledge and lower sweetener knowledge was associated with higher added sweetener consumption, even when controlling for SDoH. Level of educational attainment has been directly linked with important health outcomes including life expectancy in the general population [23]. In the United States, across racial/ethnic backgrounds, adults with lower educational attainment are more likely to report worse health outcomes [24]. Higher educational attainment is often associated with higher earning employment opportunities offering health benefits, which may positively impact one’s ability to purchase more nutritious food [25, 26]. Also, individuals with higher educational attainment are more likely to have higher health literacy and understanding of recommendations regarding nutrition and healthy dietary behaviors. To our knowledge, no prior studies have accounted for educational attainment when designing dietary modification interventions in PLWH. Nutrition interventions may be more effective if customized to account for educational attainment to facilitate successful comprehension of instructions and resources to modify added sweetener consumption.

Co-morbidities, including overweight or obesity, diabetes, and CVD, are common in many PLWH, owing to traditional and non-traditional factors associated with HIV infection [27, 28]. BMI associated with added sweetener consumption in this cohort. Given what is known about the potential effects of added sweeteners on cardiometabolic health in PLWH [9], establishing lifestyle modification interventions that include education on added sweetener intake to successfully modify risk in PLWH is important. Also, existing nutrition-based lifestyle modification interventions for PLWH may have not been developed based on evidence specifically derived from PLWH, including pertinent data on SDoH that can impact behavior change and knowledge on healthy eating. Our findings suggest this is an important consideration when establishing feasible and successful nutritional interventions to meet the unique needs of PLWH.

Reduced access to fresh fruits and vegetables associated with increased added sweetener consumption. This finding may imply that respondents with decreased access to fruits and vegetables may be replacing more nutritious, lower calorie foods with less nutritious, more calorie-dense foods with added sweeteners. Reduced intake of fresh fruits and vegetables in the general population has been correlated with the development of several non-communicable diseases, including CVD [29–31]. Palar et al. evaluated an intervention that aimed to provide healthy food and snacks to PLWH and type 2 diabetes with food insecurity. This intervention resulted in improved consumption of healthy fats, fresh fruits, and vegetables, and decreased depression and binge drinking in PLWH [32]. These findings highlight how dietary interventions designed to improve access to healthy foods may effectively reduce health complications. Many PLWH endure the burden of multi-morbidity and disparate social challenges that often stem from limited financial resources [12]. Creating structural changes that improve access to healthy foods, including fresh fruit and vegetables, among PLWH may help decrease intake of added sweeteners, enhance engagement in health-promoting dietary patterns, and decrease the incidence of comorbidities in this population.

Many campaigns to educate and increase awareness of the deleterious effects of added sweeteners and help curtail the obesity epidemic have been launched in the general population by U.S. and state-based agencies [33, 34]. To our knowledge, a study evaluating the effectiveness of these campaigns on reducing added sweetener intake in PLWH has not been published. Thus, it is difficult to ascertain whether existing interventions intended to reduce added sweetener consumption are effective or need to be tailored for PLWH. If an intervention were to be designed to improve added sweetener knowledge and reduce added sweetener consumption in PLWH exclusively, findings from this study support that it should be: 1. Written and presented in plain language and piloted among PLWH (including robust enrollment of women) from differing educational backgrounds so it can be adapted to meet the needs of all levels of educational attainment; 2. Culturally sensitive for racially and ethnically diverse individuals; 3. Comprised of dietary recommendations that are accessible to income disparate PLWH living in varied community settings; and 4. Inclusive of content that describes the relationship between added sugar consumption and obesity, and the association of obesity with increased risk for the development of CVD and diabetes—conditions that are already prevalent in many PLWH.

### Limitations

This study used an online survey to evaluate added sweetener knowledge and consumption among a large geographically diverse sample of PLWH. While this method was appropriate to achieve the aims of this investigation, there are limitations. First, as with all survey data that is dependent upon participant self-report, responses are subject to the potential for recall bias and social desirability bias. Although the study advertisement was targeted to PLWH and individuals needed to attest to being HIV-positive, it cannot be confirmed that all respondents were diagnosed with HIV. In addition to online advertisements, adjunctive recruitment methods including printed flyers and post cards were distributed at community HIV organizations and health centers. However, study participation was limited to those who could access the survey via the internet which may have led to selection bias, resulting in a respondent cohort not generalizable to all PLWH in the US. The study did not include a comparison group of individuals without HIV, and this limited the ability to decipher whether our findings among PLWH are unique when compared to individuals without HIV. Lastly, significant effort was extended to advertise and partner with local and national HIV organizations to ensure equal respondent representation from diverse racial/ethnic backgrounds, sex, income, and educational levels. While the number of women enrolled in our study is higher than that of other U.S.-based HIV studies, our overall study sample—albeit representative of participant demographics in other HIV studies [35, 36]—had limited diversity of sex, race/ethnicity, and other sociodemographic variables, which limits the generalizability of findings.

## Conclusions

This novel survey study illustrates knowledge and consumption of added sweeteners among a cohort of 900 PLWH living in the United States. Importantly, findings expose distinct sociodemographic factors including sex, race, educational attainment, and aspects of food security that associate with lower added sweetener knowledge and higher consumption in PLWH. These findings support the establishment of nutrition-based lifestyle interventions that are inclusive of added sweetener education and tailored to meet diverse demographic, educational, and social needs of PLWH. Given that increasing rates of cardiometabolic indices are observed in PLWH, and more recently among those taking integrase strand transfer inhibitors [37] our results also have public health relevance. Specifically, findings emphasize the need for policies aimed to mitigate barriers to healthy food access to improve overall nutritional intake, health, and quality of life among PLWH.

## Supplementary Information

Below is the link to the electronic supplementary material.

Supplementary file1 (TIF 155 kb)

Supplementary file2 (DOCX 36 kb)

Supplementary file3 (TIF 87 kb)

## Data Availability

All data are available from the corresponding author upon request.
