# Supplementary figures and images for: The Association of Distinct Social Determinants of Health with Added Sweetener Knowledge and Consumption in a U.S. Sample of People Living with HIV
Source: AIDS Behav. 2021 Nov 3;26(5):1552–61. doi: 10.1007/s10461-021-03508-1 (PMC9001547; doi:10.1007/s10461-021-03508-1)

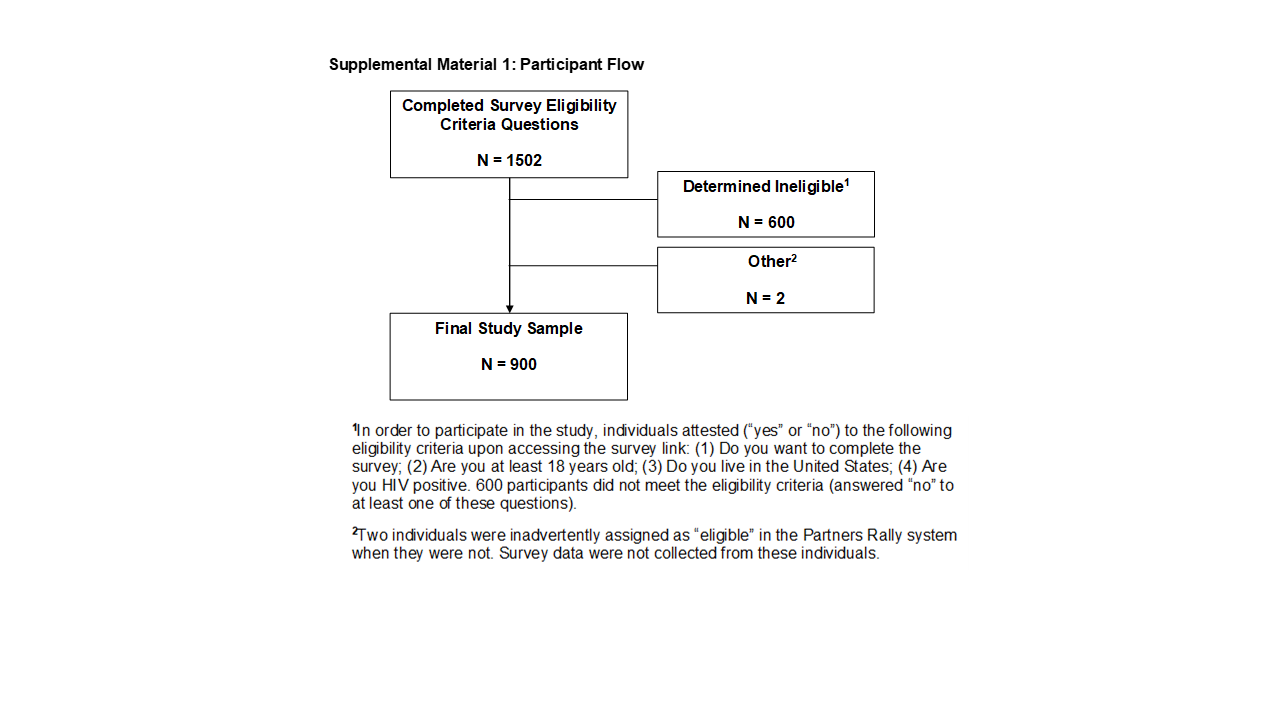

Supplement: Supplementary file 1 — Supplementary file1 (TIF 155 kb) [file 10461_2021_3508_MOESM1_ESM.tif]

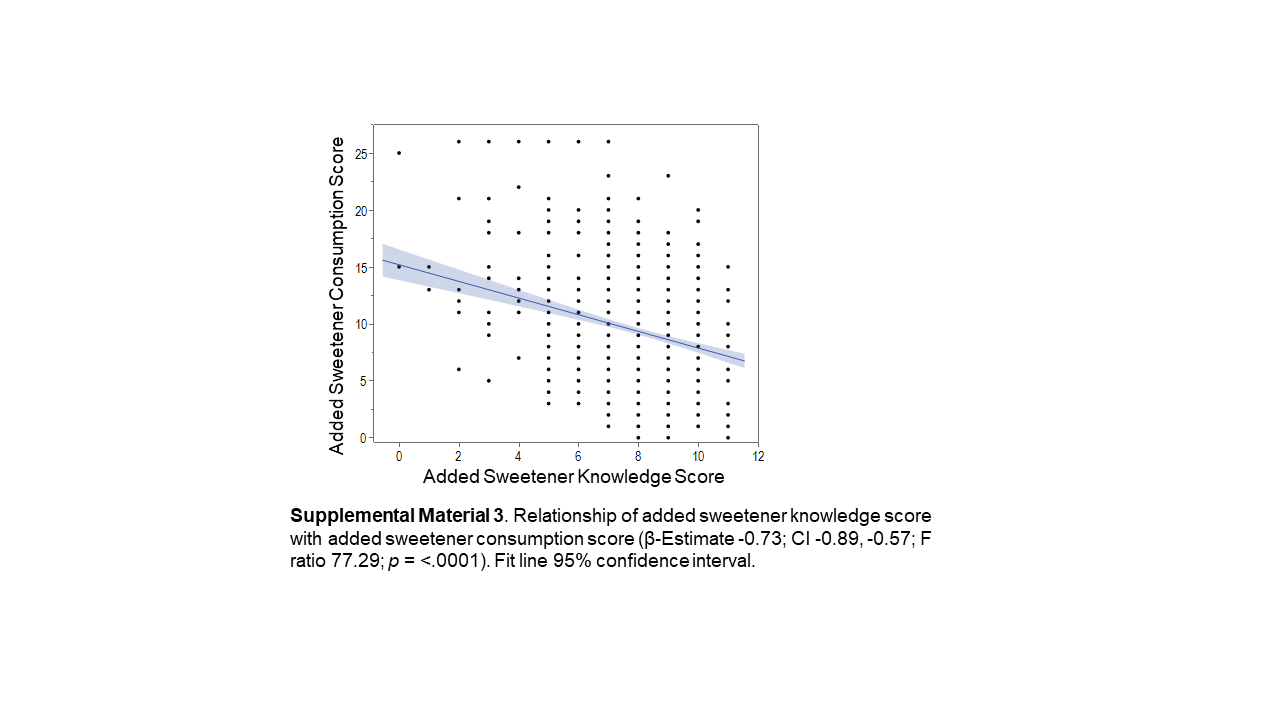

Supplement: Supplementary file 3 — Supplementary file3 (TIF 87 kb) [file 10461_2021_3508_MOESM3_ESM.tif]
