# Supplementary material for: The Association of Distinct Social Determinants of Health with Added Sweetener Knowledge and Consumption in a U.S. Sample of People Living with HIV
Source: AIDS Behav. 2021 Nov 3;26(5):1552–61. doi: 10.1007/s10461-021-03508-1 (PMC9001547; doi:10.1007/s10461-021-03508-1)
Supplement: Supplementary file 2 — Supplementary file2 (DOCX 36 kb) [file 10461_2021_3508_MOESM2_ESM.docx]

**Supplemental Material 2: Survey Content**

Introduction:

We are doing a study about sweeteners in the diet, when we use the word sweetener, we are talking about different types of sugars and artificial sweeteners. We would like to find out what people with HIV know about sweeteners, eating patterns of sweeteners, and how sweeteners may affect health. Identifiable information will not be collected during this survey. Your answers to this survey are voluntary and will be kept confidential. This survey will take about 20 minutes, please answer every question to the best of your knowledge.

**SCREENING QUESTIONS**

1. Would you like to complete this survey (By clicking yes to the question below you are consenting to proceed in the study).
   1. Yes
   2. No
2. Is English your primary language?
   1. Yes
   2. No
3. Are you at least 18 years old?
   1. Yes
   2. No
4. Do you currently live in the United States?
   1. Yes
   2. No
5. Are you HIV positive?
   1. Yes
   2. No

**Demographic, Social, and Clinical Questions**

***Please select one of the most appropriate responses for the questions below.***

1. What is your sex, are you:
   1. Male
   2. Female
2. What is your gender, are you:
3. Male
4. Female
5. Transgender Female
6. Transgender Male
7. Gender Queer
8. Gender Variant or Gender Non-Conforming
9. Other
10. How old are you? _______
11. What is your race?
12. White
13. Black or African American
14. Asian
15. Native Hawaiian or other Pacific Islander
16. American Indian or Alaskan
17. More than one race
18. Other
19. Are you Hispanic Latino?
20. Yes
21. No
22. What is your highest level of education?
23. Elementary school
24. Middle school/Junior high school
25. High school or GED
26. Associate degree
27. Undergraduate Degree (Bachelor’s degree)
28. Graduate Degree (Master’s degree)
29. Doctorate Degree
30. Are you currently:
31. Single
32. In a relationship
33. Married
34. Divorced
35. Separated
36. Widowed
37. Do you have health insurance?

A. Yes

B. No

1. Can you understand the health information you get from your doctor, nurse, or clinic?

A. Yes

B. No

1. Which best describes your yearly income?
2. $0 dollars
3. $1 to $9,999
4. $10,000 to $24,999
5. $25,000 to $49,999
6. $50,000 to $74,999
7. $75,000 to $99,999
8. $100,000 to $149,999
9. $150,000 and greater
10. Do you have stable housing?
11. Yes
12. No
13. What is your zip code? ________
14. **In the last year**, have you needed to see a doctor but could not because you didn’t have enough money?

A. Yes

B. No

1. **In the last year**, did you ever eat less than you felt you should because there wasn’t enough money for food?

A. Yes

B. No

1. Do you have access to fresh fruits and vegetables?
   1. All of the time
   2. Some of the time
   3. Never
2. Do you have a place to store and cook food?
   1. All of the time
   2. Some of the time
   3. Never
3. What **year** were you diagnosed with HIV? _______
4. Is your HIV viral load:

A. Detectable

B. Undetectable

1. Do you take medicine for your HIV?

A. Yes

B. No

C. I am supposed to take medicine, but I do not

1. **On average**, how many days per week would you say that you missed at least 1 dose of your HIV medications?
2. Everyday
3. 4-6 days/week
4. 2-3 days/week
5. Once a week
6. Less than once a week
7. Have you ever been told by a doctor that you have (Please select all that apply):
8. High blood pressure
9. High cholesterol
10. Heart disease
11. A heart attack
12. Diabetes (other than during pregnancy)
13. Prediabetes
14. Cancer
15. Asthma
16. Obesity (overweight)
17. How tall are you? ______ ft ______ inches
18. How much do you weigh? ______ pounds (lbs)

**QUESTIONS ABOUT SWEETENER KNOWLEDGE AND CONSUMPTION**

1. Where is the sugar amount located on a food or drink?

1. On the list of ingredients
2. Inside the package or drink container
3. On the Nutrition Facts label (also known as the nutrition label)
4. Sugar content is not available on packaged foods or beverages
5. Please read the list of sweeteners, and **FOR EACH ONE**, click the box if you think it is **natural, added, artificial sweeter**, or select “don’t know” if you are not sure.

|  | Natural Sugar | Added Sugar | Artificial Sweetener | Don’t Know |
| --- | --- | --- | --- | --- |
| Is Honey a: |  |  |  |  |
| Is Molasses a: |  |  |  |  |
| Is Aspartame (like Nutrasweet^®^) a: |  |  |  |  |
| Is Sucrose a: |  |  |  |  |
| Is Saccharin (like Sweet’n Low^®^) a: |  |  |  |  |
| Is Fructose a: |  |  |  |  |
| Is High fructose corn syrup a: |  |  |  |  |

1. Which foods ***may*** contain added sweeteners?
2. Fruit drinks
3. Yogurt
4. Cereals
5. Chocolate Milk
6. Ketchup
7. All of the above
8. Do you think your HIV **medicines** affect the amount of sweets you eat?
9. Yes
10. No
11. Do you eat more sweets when your **HIV viral load** is high?
12. Yes
13. No
14. Do you think **HIV infection** affects the amount of sweets you eat or drink?
15. Yes
16. No
17. What do you ***usually*** sweeten your coffee or tea with?
    1. Artificial Sweetener (Like Sweet’n Low^®^ or NutraSweet^®^)
    2. Natural Sweetener (like sugar or honey)
    3. Honey
    4. I don’t *usually* sweeten my coffee or tea
18. **IN AN AVERAGE WEEK,** how often do you (please click the box that best describes your habits):

|  | Usually/Often | Sometimes | Rarely/Never |
| --- | --- | --- | --- |
| *DRINKS* |  |  |  |
| Drink juice (like orange, apple, grapefruit, grape juice, etc)? |  |  |  |
| Drink other fruit drinks (like fruit punch or Kool-Aid^®^)? |  |  |  |
| Drink NON-DIET soft drinks, soda or pop? |  |  |  |
| Drink DIET OR SUGAR-FREE fruit or soft drinks, soda, or pop? |  |  |  |
| Drink sports drinks (like Gatorade^®^ or Powerade^®^)? |  |  |  |
| Drink energy drinks (like Red Bull^®^ or Monster^®^)? |  |  |  |
| Drink DIET OR SUGAR-FREE sports drinks or energy drinks? |  |  |  |
| Drink more than 1-2 alcoholic drinks A DAY? (One drink = 12 oz. beer, 5 oz. Wine, one shot of hard liquor or mixed drink with 1 shot) |  |  |  |
| *FOODS* |  |  |  |
| Eat jam, jelly, or honey on bagels, muffins, rolls, or crackers? |  |  |  |
| Eat ice cream, ice cream bars, pudding, frozen yogurt, or sherbet? |  |  |  |
| Eat cake, cookies, brownies, donuts, muffins, or dessert breads? |  |  |  |
| Eat fruit crisp, cobbler, pie, or pop tarts? |  |  |  |
| Eat chocolate candy (like chocolate bars, fudge, peanut butter cups)? |  |  |  |
| Eat other candy (like gummies, hard candy, sour candy)? |  |  |  |

1. **True or false,** the American Heart Association recommends **women** limit how much sugar they eat to 6 teaspoons per day and **men** to 9 teaspoons per day.
   1. True
   2. False
2. Too much added sugar is known to increase your risk for:
   1. Obesity (overweight)
   2. Type 2 diabetes
   3. High blood pressure
   4. High cholesterol
   5. All of the above

**References**

The questions in this survey were established based on the investigator’s prior research, clinical experience and adapted from the surveys referenced below after participant feedback from a focus group evaluating our survey content.

Lin, C.-T.J., Zhang, Y., Carlton, E.D., Lo, S.C. 2014 FDA health and diet survey. Center for Food and Applied Nutrition Food and Drug Administration, May 6, 2016; 1-43.

Mannheimer, S.B., Mukherjee, R., Hirschhorn, L.R., Dougherty, J., Celano, S.A., et al. The CASE adherence index: a novel method for measuring adherence to antiretroviral therapy. AIDS Care, October 2006; 18(7): 853-861.

National Health and Nutrition Examination Survey (NHANES) Food Frequency Questionnaire; Created by the National Institutes of Health and National Cancer Institutes. Available at: <https://www.cdc.gov/nchs/data/nhanes/nhanes_03_04/tq_fpq_c.pdf>

Rapid Eating Assessment for Patients (REAP). 2005 Institute for Community Health Promotion, Brown University, Providence, RI. Available at: <https://snaped.fns.usda.gov/library/materials/rapid-eating-assessment-participants-reap-s>

The Social Needs Screening Toolkit. Health Leads: <https://healthleadsusa.org/>

Tierney, M., Gallagher, A.M., Giotis, E.S., Pentieva, K. An online survey on consumer knowledge and understanding of added sugars. Nutrients, 2017; 9(37).
